# Elements of Chemistry, Including the History of the Imponderables and the Inorganic Chemistry of the Late Edward Turner, M.D., F.R.S.L.&E.

**Published:** 1846-06

**Authors:** 


ART. XIII.—*Elements of Chemistry, including the history of the Imponderables and the Inorganic Chemistry of the late* EDWARD TURNER, M. D., F.R.S.L.&E. Seventh edition. *And the Outlines of Organic Chemistry*, By WILLIAM GREGORY, M.D , &c., Professor of Chemistry in the University of Edinburgh. *With Notes and Additions.* By JAMES B. ROGERS, M.D., Professor of General Chemistry, Franklin Institute; Lecturer on Medical Chemistry, &c.; and ROBERT E. ROGERS, M.D., &c., Professor of Chemistry and Materia Medica, University of Virginia, &c. Philadelphia: Thomas, Cowperthwaite & Co. 1846. 8vo. pp. 848.

As we remarked of physiology, just now, it is emphatically true of chemistry, that it requires a continual posting up, in order that men may know what new triumphs it has been achieving. This work of Dr. Turner's was long the decided favorite in this country of all the treatises on Chemistry. So long as the author lived he continued to retouch it, issuing new editions from time to time, which embraced all the discoveries and improvements in the science. When his lamented death occurred, his "Elements" gradually declined, or were partially superseded by more modernized works. From this temporary eclipse it is the purpose of the editors to recover it, and we have no doubt their effort will be attended with success. All that this admirable system needed was a periodical revision, keeping it up with the march of chemical science. Having now been advanced to that point, it will not fail to resume its place at the head of the class of chemical text-books.

We received a copy of this valued work from the publishers through our enterprising booksellers, Messrs. Morton & Griswold.

